# A Point Mutation in p190A RhoGAP Affects Ciliogenesis and Leads to Glomerulocystic Kidney Defects

**DOI:** 10.1371/journal.pgen.1005785

**Published:** 2016-02-09

**Authors:** Katherine Stewart, Yaned Gaitan, Maxwell E. R. Shafer, Lamine Aoudjit, Di Hu, Richa Sharma, Mathieu Tremblay, Hidetaka Ishii, Michael Marcotte, Daniela Stanga, You Chi Tang, Sami Kamel Boualia, Alana H. T. Nguyen, Tomoko Takano, Nathalie Lamarche-Vane, Silvia Vidal, Maxime Bouchard

**Affiliations:** 1 Goodman Cancer Research Centre and Department of Biochemistry, McGill University, Montreal, Quebec, Canada; 2 Department of Medicine, McGill University Health Centre, Montreal, Quebec, Canada; 3 Department of Anatomy and Cell Biology, McGill University, and Research Institute of the McGill University Health Centre, Montreal, Quebec, Canada; 4 Department of Human Genetics and Department of Microbiology and Immunology, McGill University, Montreal, Quebec, Canada; Seattle Children's Research Institute, UNITED STATES

## Abstract

Rho family GTPases act as molecular switches regulating actin cytoskeleton dynamics. Attenuation of their signaling capacity is provided by GTPase-activating proteins (GAPs), including p190A, that promote the intrinsic GTPase activity of Rho proteins. In the current study we have performed a small-scale ENU mutagenesis screen and identified a novel loss of function allele of the p190A gene *Arhgap35*, which introduces a Leu1396 to Gln substitution in the GAP domain. This results in decreased GAP activity for the prototypical Rho-family members, RhoA and Rac1, likely due to disrupted ordering of the Rho binding surface. Consequently, *Arhgap35-*deficient animals exhibit hypoplastic and glomerulocystic kidneys. Investigation into the cystic phenotype shows that p190A is required for appropriate primary cilium formation in renal nephrons. P190A specifically localizes to the base of the cilia to permit axoneme elongation, which requires a functional GAP domain. Pharmacological manipulations further reveal that inhibition of either Rho kinase (ROCK) or F-actin polymerization is able to rescue the ciliogenesis defects observed upon loss of p190A activity. We propose a model in which p190A acts as a modulator of Rho GTPases in a localized area around the cilia to permit the dynamic actin rearrangement required for cilia elongation. Together, our results establish an unexpected link between Rho GTPase regulation, ciliogenesis and glomerulocystic kidney disease.

## Author Summary

Glomerulocystic kidney disease occurs either in isolation or in combination with other cystic diseases. To date, the paucity of mouse models have impeded our progress in understanding the molecular mechanisms leading to glomerular cyst development. Using an ENU mutagenesis approach, we present here a novel mouse model of glomerular cyst formation caused by a point mutation in p190A RhoGAP (*Arhgap35*), which is associated with aberrant primary ciliogenesis. The primary cilium is a microtubule-based signalling center involved in cell differentiation and homeostasis. A role for the actin cytoskeleton in ciliogenesis has recently emerged, but the underlying regulatory mechanisms remain poorly understood. In this study we identify a requirement for the local modulation of Rho GTPase by p190A RhoGAP during primary cilium formation, which constitutes the first demonstration of ciliogenic regulation by RhoGAP proteins. Together, this work identifies deficiencies in actin cytoskeletal dynamics as an underlying cause for ciliary and glomerulocystic malformations.

## Introduction

The dynamic regulation of actin polymerization is crucial for cell physiology and closely associated with cellular processes such as migration, survival, polarization and cytokinesis [1,2,3]. Among the main regulators of actin dynamics are the Rho family of GTPases, including RhoA, Rac1 and Cdc42, which act as molecular switches that cycle between the active, GTP-bound and inactive, GDP-bound forms [1,4,5,6]. The critical regulation of this cycle is provided by three classes of molecules: guanine nucleotide exchange factors (GEFs) exchange GDP for GTP, thereby activating Rho-family proteins, whereas GTPase activating proteins (GAPs) accelerate the intrinsic GTP hydrolytic activity of the Rho-family to attenuate signaling [6,7,8]. In addition, guanine nucleotide dissociation inhibitors (GDIs) bind to Rho GTPases and keep them in their inactive state, thereby preventing downstream effector activation. In spite of their extensive characterization in vitro and in cell culture models, the role of RhoGAPs is still poorly understood during animal development and associated diseases.

The RhoGAP protein p190A (*Arhgap35*, *Grlf1*) is a potent regulator of Rho GTPases. It is composed of a N-terminal GTPase domain necessary for its activity, a series of conserved FF domains, and a C-terminal RhoGAP domain [9,10,11]. p190A RhoGAP activity has been observed in vitro against the prototypical Rho GTPases, namely Rac1, Cdc42 and RhoA, with the strongest activity on the latter [12,13,14]. In line with this observation, the cellular activity of p190A seems primarily directed against RhoA [13]. Gene inactivation studies in the mouse identified an important role for p190A in neural tube closure, eye morphogenesis and mammary gland branching morphogenesis, differentiation and cell adhesion [15,16,17,18]. Little is known, however, about the cellular role of p190A in tissue morphogenesis, notably in the urogenital system.

In higher vertebrates, the formation of the definitive kidneys and urinary tracts commences with the budding and branching of the ureter tree by signals from the adjacent metanephric mesenchyme [19,20,21]. Conversely, nephron formation is initiated by signals from the ureter tip to induce metanephric mesenchyme condensation and epithelialization into renal vesicles, which proceeds through comma and s-shaped bodies [22]. Invasion of endothelial cells in the cleft of the s-shaped body results in the formation of the vascular and mesangial aspects of the glomerulus, while the epithelial component of the s-shaped body gives rise to nephron tubules, Bowman’s capsule and the podocyte lineage that surround the vasculature and act as a filtration barrier [22,23]. In the adult kidney Rho family GTPases are required to maintain the glomerular slit barrier formed by podocytes. Alterations in the activity of RhoA, Rac1 and Cdc42 in differentiated podocytes leads to proteinuria and focal segmental glomerulosclerosis associated with foot process effacement [24,25,26,27,28,29]. Despite the relatively well-characterized role of the Rho proteins in adult glomerular maintenance, little is known about Rho GTPases and their regulators during nephron development.

Polycystic kidney disease is one of the most common renal pathologies [30]. While the underlying mechanism of cyst formation remains somewhat unclear, pathogenesis is often associated with defects in ciliogenesis or cilia function [31,32]. Primary cilia are microtubule-based cell surface projections that serve as signalling platforms for a number of developmentally important pathways [33]. Initiation of ciliogenesis requires vesicular trafficking of the mother centriole to, and subsequent docking with, the plasma membrane [34,35]. The docked centriole, known as the basal body, nucleates elongation of the ciliary axoneme by serving as a scaffold for ciliary-cargo containing vesicles [34,35]. Elongation of the axoneme occurs via microtubule trafficking by intraflagellar proteins attached to kinesin and dynein motors [36,37]. Interestingly, recent evidence suggests that regulation of actin cytoskeletal dynamics is required for the appropriate docking of the basal body and subsequent trafficking of ciliary vesicles [38,39,40,41,42,43].

Glomerulocystic disease is a subtype of polycystic kidneys that exhibit primarily glomerular cysts, characterized by severe dilation of Bowman’s capsule and progressive glomerular atrophy [44]. It manifests in both familial and sporadic forms, and often occurs as a component of other genetic syndromes [44]. There are very few mouse models of glomerulocystic kidney disease [45,46], and although the two reported models implicate loss of primary cilia in cyst formation, the regulatory pathways involved are unclear. Here we performed a small-scale *N-*ethyl-*n*-nitrosourea (ENU) mutagenesis screen [47] to identify new regulators of urogenital system development and disease. We identify a mutant strain with hypoplastic and glomerulocystic kidneys, resulting from a loss-of-function mutation in p190A RhoGAP (*Arhgap35*). We further demonstrate a crucial role for p190A in modulating actin dynamics during ciliogenesis.

## Results

**Fig 1:**
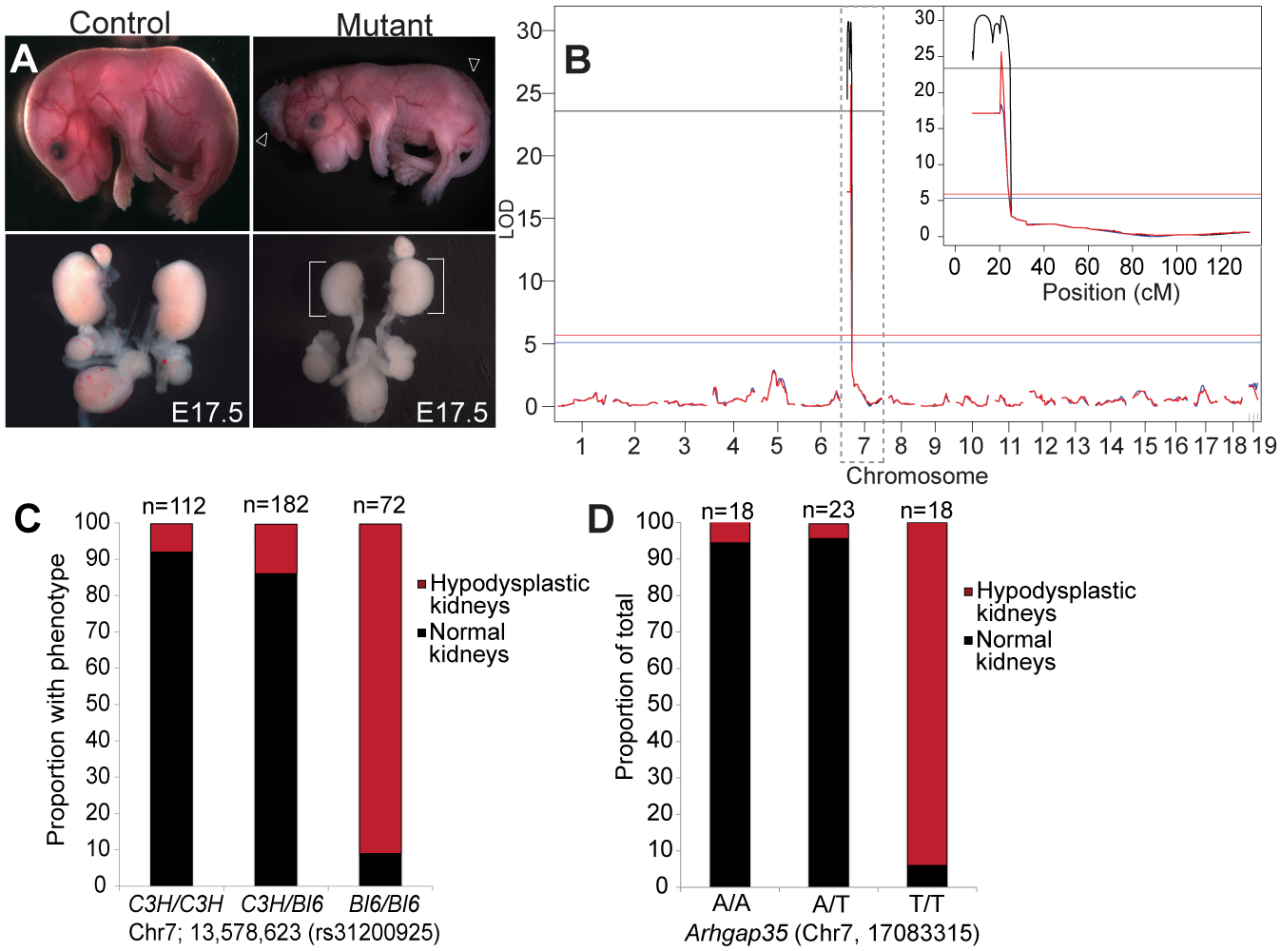
Mapping of ENU-generated mutation identifies a point mutation in *Arhgap35* (p190A RhoGAP) that results in kidney hypodysplasia. *(A)* The ENU-mutant line D34 shows exencephaly and spina bifida (open arrowheads), concomitant with kidney hypodysplasia (brackets) at E17.5. *(B)* Single nucleotide polymorphism (SNP)-array screening of 76 embryos identifies a single significant region on chromosome 7 associated with kidney hypodysplasia using algorithms EM [black], Haley-Knott [blue] and Multiple Imputation [red]. Significance threshold is indicated by horizontal line for each algorithm. *(C)* Restriction fragment length polymorphism analysis between the wild type C3H/HeNCrl and mutated C57BL/6J strains using marker rs31200925 (N = 346 embryos) confirms the association with kidney hypodysplasia. *(D)* Sanger sequencing of the *Arhgap35* A to T sequence variant identified by exome sequencing reveals a strong association with kidney hypodysplasia (N = 59 embryos).

**Fig 2:**
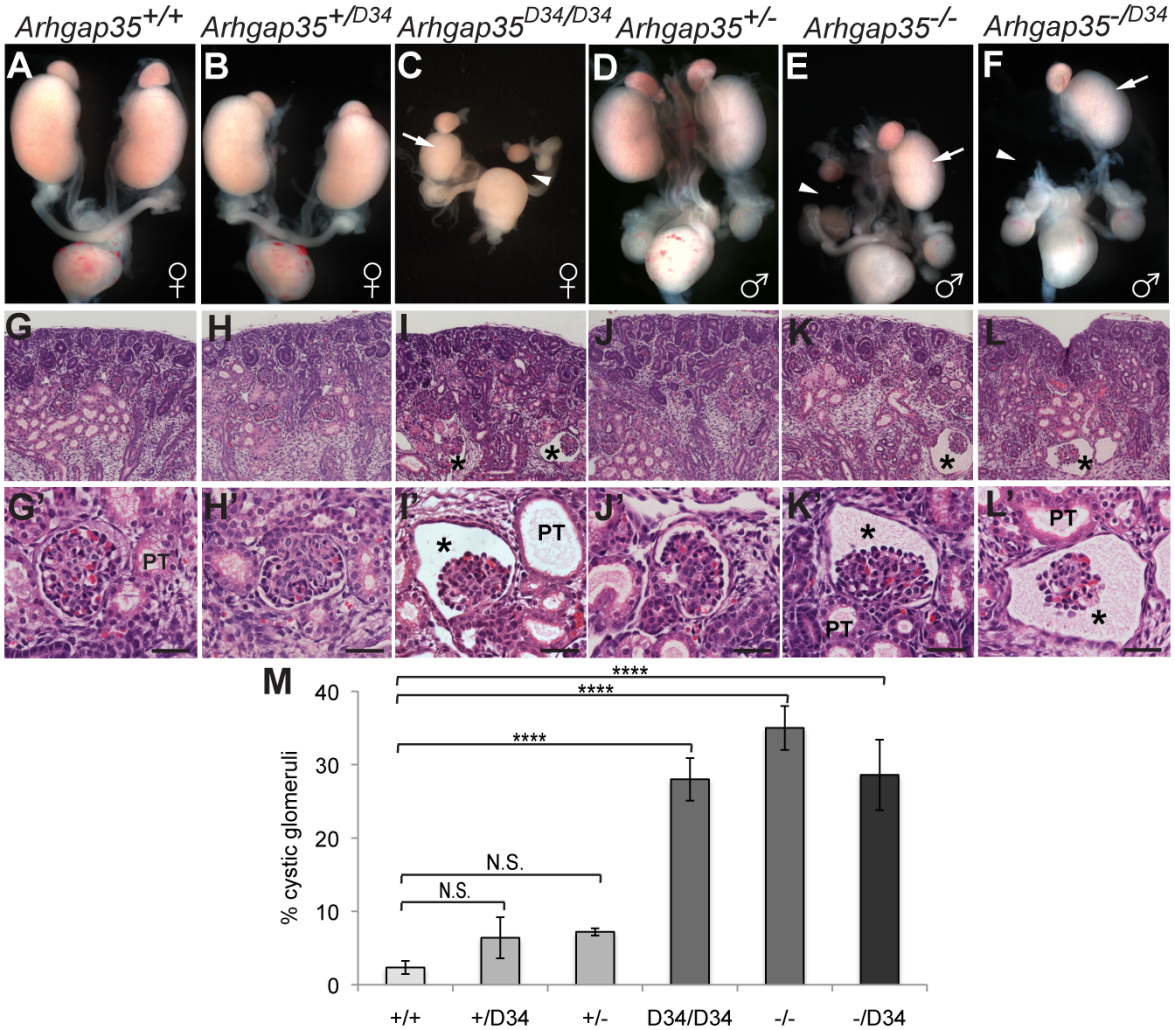
Crosses between *Arhgap35*^*D34*^ and an independent *Arhgap35* mutant allele demonstrate non-complementation, resulting in hypoplastic and glomerulocystic kidneys. *(A-F)* Whole urogenital systems dissected at E17.5. In contrast to normal sized kidneys in control animals (panels A,B,D), *Arhgap35*^*D34/D34*^(C), *Arhgap35*^*-/-*^(E), and *Arhgap35*^*-/D34*^(F) kidneys exhibit hypoplasia (arrows) or agenesis (arrowheads). *(G-L)* Hematoxylin and Eosin stained sections (20X) show large glomerular cysts (asterisks), accompanied by occasional proximal tubule [PT] dilation in *Arhgap35*-deficient animals (I,K,L). *(G’-L’)* Higher magnification (63X) images show that glomerular architecture is largely preserved in cystic glomeruli. Scale bars, 20μm *(M)* Kidneys from the *Arhgap35*^*D34/-*^ allelic series show a similar proportion of cystic glomeruli, counted from H&E stained sections. *p<0.05, **p<0.01, ***p<0.005, ****p<0.001 (one-way ANOVA)

To uncover novel regulators of mammalian urogenital system development, we undertook a recessive ENU mutagenesis approach in which a total of 28 G1 C57BL/6J males (progeny of ENU treated G0 males) were out-crossed to wild type C3H/HeNCrl females to generate G2 mice (S1 Fig). Phenotypic screening was performed on E17.5 embryos from G2 females mated to their respective G1 males. Of the G1 lines tested, line D34 consistently harboured severe kidney defects, including hypoplastic kidneys with glomerular cysts (hypodysplastic kidneys) without associated ureter obstruction and a low penetrance of duplex urogenital systems (Figs 1A, 2C, 2I and 2I’). Interestingly, approximately half of embryos with urogenital system anomalies also exhibited neural tube closure defects, including exencephaly and spina bifida (Fig 1A). Homozygous D34 mutation results in perinatal lethality, with few mutant animals surviving to weaning age.

Single nucleotide polymorphism (SNP)-based linkage analysis was undertaken to identify the genetic locus associated with kidney hypodysplasia in affected embryos. The characterization of 76 samples identified a single peak of approximately 18 cM on chromosome 7 (rs3675839-rs31924991) (Fig 1B). Additional linkage analysis was performed by restriction length fragment polymorphism (RFLP) on genomic DNA from 346 embryos using markers rs31200925 and rs31924991. This analysis revealed a strong association between hypodysplastic kidneys with the proximal region of chromosome 7 (Fig 1C). Given that there are over 170 genes contained within this chromosomal region, we performed whole exome sequencing on 5 affected embryos to narrow down possible causative mutations. We found a single candidate mutation (A to T) in *Arhgap35* (p190A RhoGAP gene), which was located within the 18cM interval and occurred in all affected embryos. To further investigate the association between the altered allele and the kidney phenotype, we Sanger-sequenced *Arhgap35* in 59 additional embryos. As expected, the vast majority of affected kidneys were homozygous for the altered *Arhgap35* allele (Fig 1D) suggesting that it is likely to be causal in the renal abnormalities of line D34.

### Genetic validation of *Arhgap35* deficiency in glomerulocystic phenotype

To address whether the renal anomalies observed in the D34 line (hereafter referred to as *Arhgap35*^*D34*^) result from a loss-of-function mutation in the *Arhgap35* gene, we obtained the previously generated *Arhgap35*^*tmjset1*^ mutant line [16] (referred to as *Arhgap35*^*-*^ mice) and generated homozygous mutant and compound heterozygous mutant animals for both alleles. Gross histological characterization of the urogenital system of *Arhgap35*^*D34/D34*^ kidneys at embryonic day 17.5 (E17.5) revealed a defined renal cortex and medulla, similar to control animals (Fig 2A, 2B, 2C, 2G, 2H and 2I). In line with this observation, differentiation markers showed no significant differences between control and mutant kidneys (S2A, S2B and S2C Fig). Remarkably, however, serial sectioning revealed that all *Arhgap35*^*D34/D34*^ kidneys contained cystic glomeruli accompanied by occasional dilation of the nearby proximal tubule (Figs 2I, 2I’, S2D and S3D). *Arhgap35*^*-/-*^ kidneys exhibited similarly penetrant renal malformations not previously described, including kidney hypoplasia and agenesis, as well as cystic glomeruli (Fig 2E, 2K, 2K’ and 2M and Table 1). As previously reported, these animals also harboured neural tube closure defects resulting in early perinatal lethality ([16], S3A and S3B Fig). Notably, compound heterozygous mice (*Arhgap35*^*D34/-*^) showed early perinatal lethality, neural tube closure defects (S3A, S3B and S3C Fig and Table 1) and a range of renal abnormalities including hypoplasia, agenesis and glomerular cysts at a similar frequency to both *Arhgap35*^*D34/D34*^ and *Arhgap35*^-/-^ embryos (Fig 2F, 2L, 2L’ and 2M, and Table 1). Together, these results demonstrate that the D34 line is a novel *Arhgap35* mutant strain and highlight the importance of p190A activity during kidney development.

**Table 1:** *Arhgap35*-deficient animals exhibit neural tube closure defects (exencephaly and spina bifida) and hypodysplastic kidneys in E17.5 embryos.

	Neural tube defects		Urogenital system defects		Number
	Normal	Open	Normal	Hypodysplastic	
***Arhgap35***^***+/D34***^ ***x Arhgap35***^***+/D34***^					
*Arhgap35*^*+/+*^	96%	4%	87%	13%	112
*Arhgap35*^*+/D34*^	91%	9%	87%	13%	196
*Arhgap35*^*D34/D34*^	46%	54%	9%	91%	78
***Arhgap35***^***+/-***^ ***x Arhgap35***^***+/-***^					
*Arhgap35*^*+/+*^	100%	0%	94%	10%	20
*Arhgap35*^*+/-*^	97%	3%	74%	26%	39
*Arhgap35*^*-/-*^	56%	44%	0%	100%	18
***Arhgap35***^***+/D34***^ ***x Arhgap35***^***+/-***^					
*Arhgap35*^*+/+*^	100%	0%	95%	5%	15
*Arhgap35*^*+/D34*^	100%	0%	94%	6%	16
*Arhgap35*^*+/-*^	97%	3%	83%	17%	29
*Arhgap35*^*D34/-*^	62%	38%	0%	100%	8

The similar penetrance of these malformations in *Arhgap35*^*D34/D34*^, in an independent *Arhgap35* mutant line, and in compound heterozygous animals confirms D34 as a *Arhgap35* mutant strain and suggests a strong hypomorphic allele.

### *Arhgap35*^*D34*^ is a loss-of-function allele

**Fig 3:**
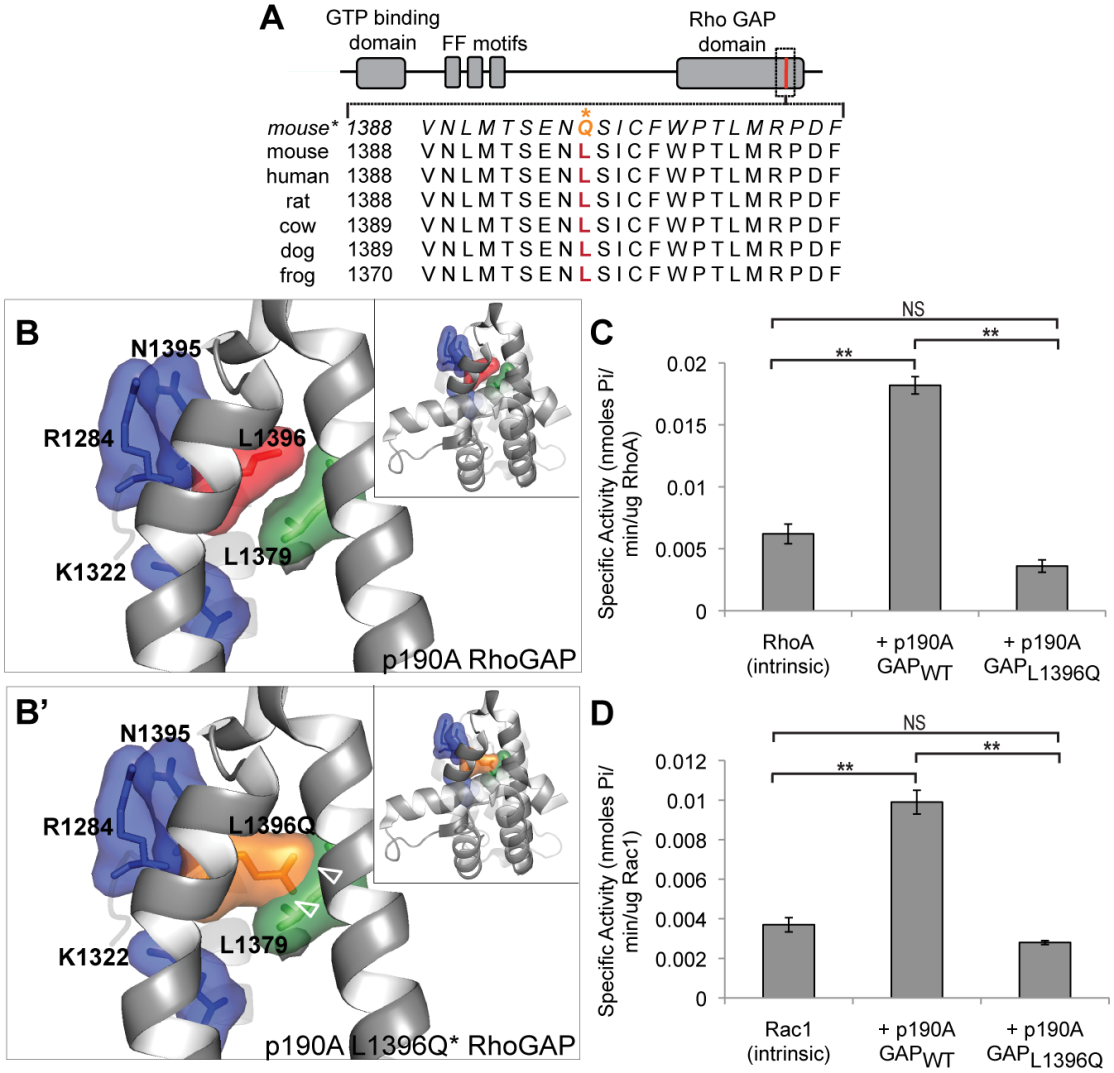
L1396Q substitution in the p190A GAP domain is a loss-of-function mutation. *(A)* Protein sequence alignment around leucine 1396 (red) shows high conservation across species. The predicted L1396Q mutation (*) is indicated in orange. *(B-B’)* Structure of the human p190A GAP domain (PDB 3FK2) residues around L1396 [red], including RhoA-interacting residues R1284, K1322, and N1395 [blue] and nearby residue L1379 [green], reveals substantial steric clashes with the substituted glutamine [L1396Q, orange](open arrowhead). Insets show the full GAP domain structure. *(C-D)* In vitro Rho-family GTPase activity assays in the absence of recombinant p190A GAP domain (intrinsic), with the wild type (p190A GAP_WT_) or point mutant (p190A GAP_L1396Q_) domains reveal a loss of GAP activity of the mutant form for recombinant RhoA (panel C) and Rac1 (panel D). *p<0.05, **p<0.01 (one-way ANOVA)

The phenotypic similarity and non-complementation between the *Arhgap35*^-^ and *Arhgap35*^*D34*^ alleles suggested that *Arhgap35*^*D34*^ is a loss-of-function mutation. The ENU-induced A to T nucleotide exchange alters amino acid leucine 1396 to glutamine in the RhoGAP domain of p190A (Fig 3A). To gain insight into the functional significance of L1396, we first examined the interspecies conservation of the residue. Interestingly, L1396 is identical across species from mice to frogs and is located in a highly conserved region (Fig 3A), suggesting an important functional requirement for this domain. We next investigated the potential structural impact of the mutation on p190A function by examining the crystallized human p190A GAP-domain structure (PDB: 3FK2) (Fig 3B and 3B’). Notably, L1396 is located on the interior of a tightly packed α-helical interface directly adjacent to the ordered surface formed by the highly conserved RhoA binding residues K1322, and N1395 and the catalytic arginine (R1284) required for p190A function [8,9,48,49] (Fig 3B). Modeling the conversion of L1396 to the bulkier glutamine reveals substantial steric clash with surrounding amino acids, irrespective of the position of the glutamine side chain (Fig 3B’). Based on this structural modeling, it is likely that the L1396Q substitution distorts the binding site on p190A, reducing its ability to act as an effective GAP.

As our genetic studies suggested a loss-of-function mutation, we predicted that it would result in reduced GTP hydrolysis by RhoA and/or Rac1, relative to wild type p190A. To examine this possibility we expressed either the wild-type GAP (p190A GAP_WT_) or D34 mutant GAP (p190A GAP_L1396Q_) domains as GST-fusion proteins for in vitro GAP activity assays. As expected, each small GTPase possessed little intrinsic GTP-hydrolytic activity alone, while addition of p190A GAP_WT_ significantly enhanced the rate of GTP hydrolysis (Fig 3C and 3D), reflecting an acceleration of Rho GTPase activity in line with previously published results [9,10,13]. Interestingly, p190A GAP_WT_ showed a distinct preference for RhoA over Rac1, reflected in the increase in specific activity (Fig 3C and 3D). Strikingly, the mutant p190A GAP_L1396Q_ protein severely impaired phosphate release for both substrates (Fig 3C and 3D), reflecting a significant reduction in GAP activity. In line with this observation, GTP-bound RhoA is enhanced in mouse embryonic fibroblasts derived from *Arhgap35*^*D34/D34*^ animals, as compared to controls (S3D Fig). Together these results confirm *Arhgap35*^*D34*^ as a loss-of-function allele likely resulting from a disruption of the p190A RhoGAP binding interface.

### P190A is required for ciliogenesis

**Fig 4:**
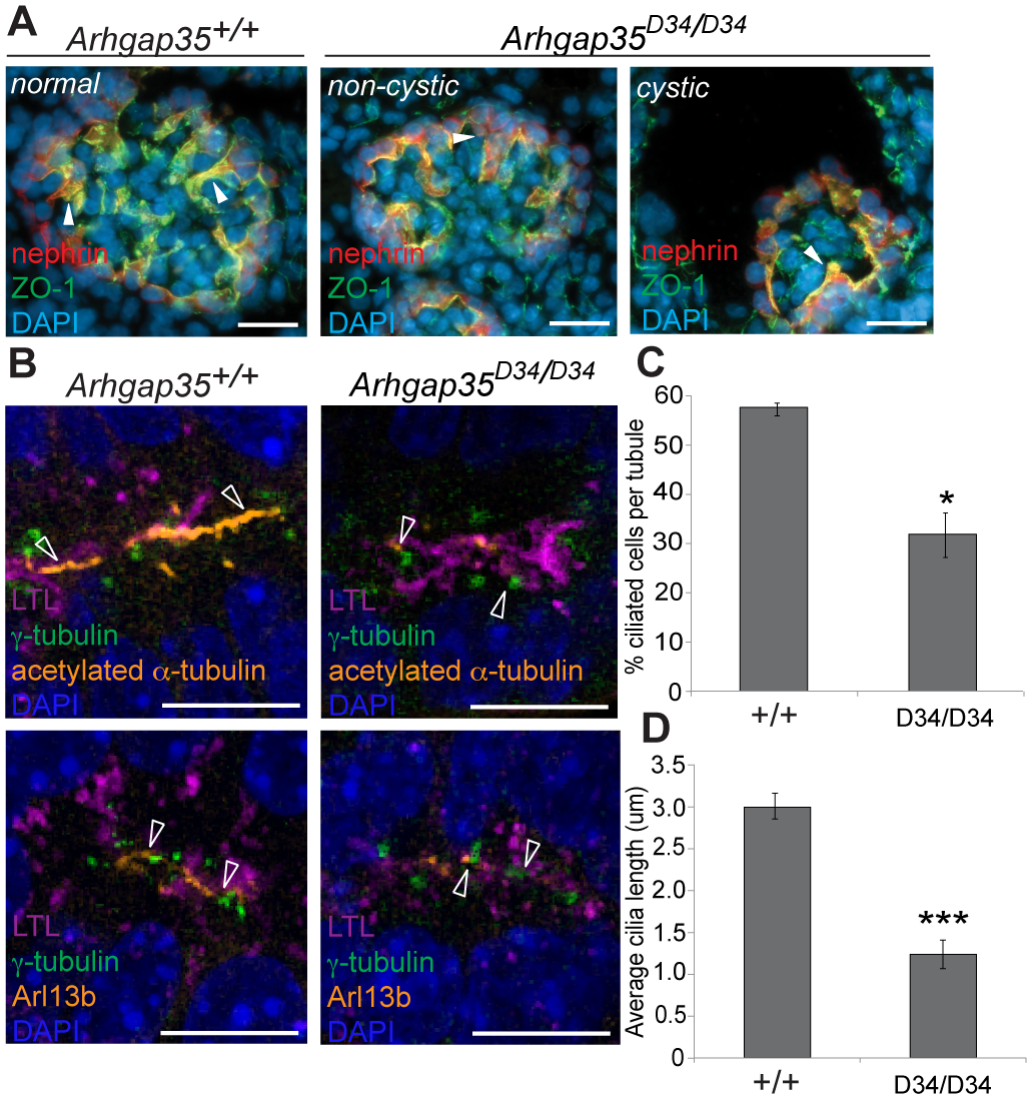
Glomerulocystic phenotype in *Arhgap35*^*D34/D34*^ is associated with a ciliogenesis defect. *(A)* Immunofluorescent staining of control and mutant glomeruli sections at E17.5 show that podocytes (marked by nephrin expression) form ZO-1^+^ tight junctions, reflective of an intact slit diaphragm (closed arrowheads). Scale bars, 10μm *(B-D)* Immunofluorescent staining for acetylated α-tubulin and Arl13b in proximal tubules (marked by Lotus Tetragonolobus Lectin; LTL)(panel B) shows a significant decrease in the number (panel C) and length (panel D) of ciliated cells in *Arhgap35*^*D34/D34*^ animals compared to wild type (open arrowheads). Scale bars, 5μm. *p<0.05, **p<0.01, ***p<0.005 (unpaired, two-tailed Student’s *t*-test)

Given the relative paucity of mouse models of glomerulocystic disease, we decided to further characterize the molecular basis of glomerular cyst formation in *Arhgap35*-deficient kidneys. We first examined the expression of the podocyte marker *Wt1*, which showed a normal progression of glomerular development and overall density in *Arhgap35*^*D34/D34*^ animals (S4 Fig). This result, together with the normal capillary loops, mesangial cells and podocytes in both cystic and normal glomeruli observed by H&E (Fig 2), indicated that the cystic phenotype is unlikely to result from defects in early glomerular development. We next asked whether *Arhgap35* mutation compromised the structure of the glomerular tuft. For this, we performed immunofluorescence staining for podocyte specific proteins, including nephrin, synaptopodin and Wt1, as well as the structural markers laminin, ZO-1 and F-actin (Fig 4A and S4 Fig). We did not observe significantly altered expression or localization of any of the markers examined in non-cystic mutant glomeruli, indicating that prior to cyst formation *Arhgap35*^*D34/D34*^ glomeruli are largely indistinguishable from controls. In addition, glomerular tufts of *Arhgap35*^*D34/D34*^ animals showed evidence of F-actin rich foot processes despite significant dilation of Bowman’s capsule (Figs 4A and S4). Together, these results suggest that the glomerulocystic phenotype of *Arhgap35*^*D34/D34*^ kidneys does not primarily result from glomerular morphogenesis defects.

We next assessed the expression of *Arhgap35* by in situ hybridization in developing kidneys. At E17.5, *Arhgap35* expression was present but low in the glomerulus, which contrasted with its high expression levels in the proximal tubule (S5 Fig). This result raised the possibility that the glomerulocystic phenotype may be initiated in proximal tubules rather than in the glomerular epithelium.

Cystic renal phenotypes are often associated with defective ciliogenesis, resulting in dysregulation of downstream signaling pathways important for maintaining kidney epithelial homeostasis [31,50,51]. To investigate the possibility of a ciliogenesis defect, we visualized cilia by immunofluorescence against acetylated-α-tubulin and Arl13b, which mark the elongated axoneme, in combination with markers of different nephron segments. Whereas primary cilia appeared normal in glomeruli (S4E and S4F Fig), both the number of ciliated cells and the average length of the cilia were drastically reduced in the proximal tubules of *Arhgap35*^*D34/D34*^ kidneys (Fig 4B, 4C and 4D). Interestingly, cilia formation appeared impaired in both s-shaped bodies and E14.5 proximal tubules of *Arhgap35*^*D34/D34*^ mutant animals (S6 Fig), suggesting that defective proximal tubule ciliogenesis occurs during nephrogenesis, prior to the onset of cystic disease.

We next examined whether these ciliogenesis defects were secondary to abnormal basal body docking to the plasma membrane. Interestingly, γ-tubulin staining revealed appropriate positioning of the basal body in both control and *Arhgap35*^*D34/D34*^ proximal tubules (Fig 4B). Hence, the defect in cilia number and length observed in *Arhgap35*-deficient proximal tubules result from impairment in axoneme extension rather than basal body migration or positioning.

### P190A acts at the primary cilium to regulate cilia elongation

**Fig 5:**
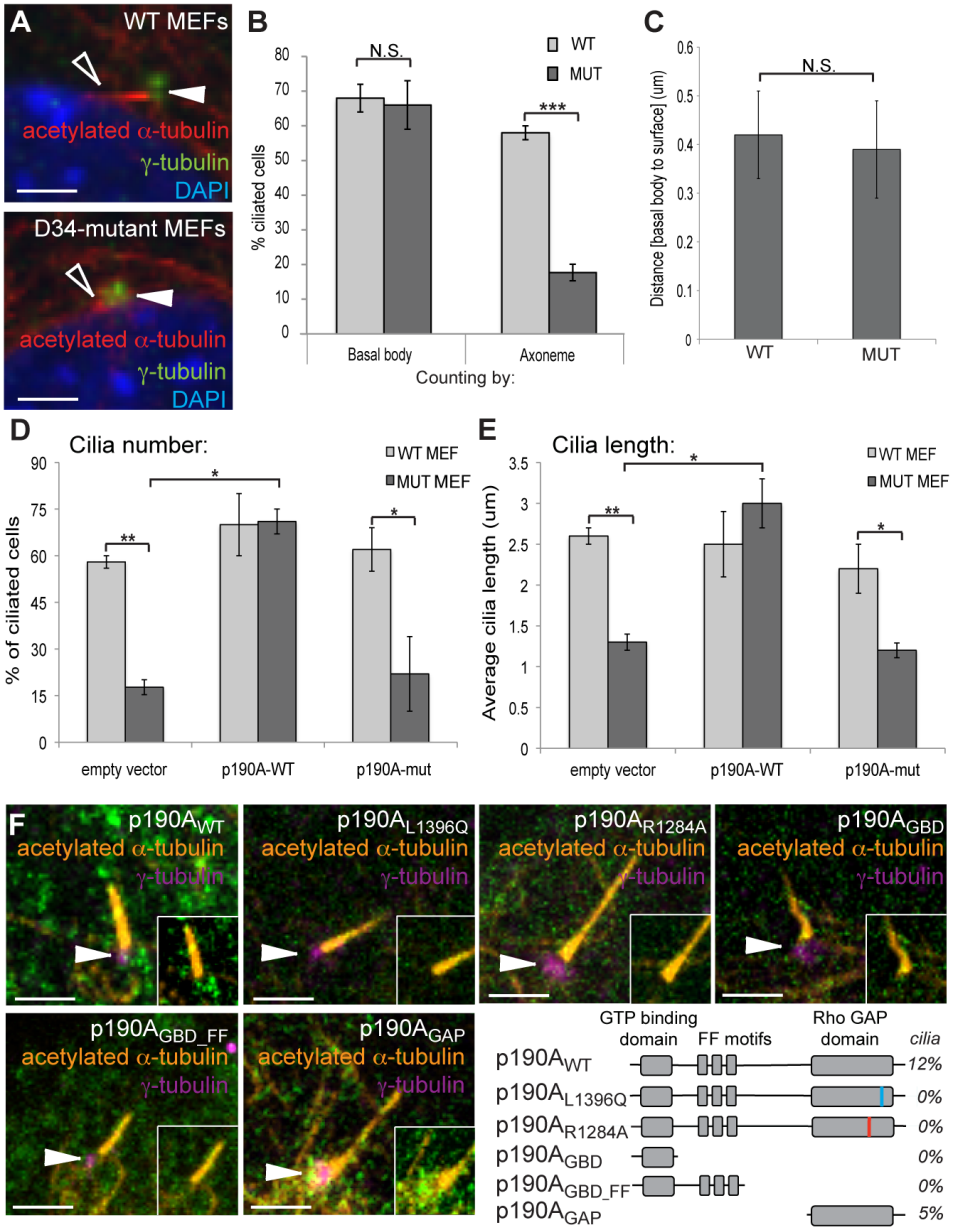
Mouse embryonic fibroblasts (MEFs) derived from *Arhgap35*^*D34/D34*^ embryos exhibit defects in cilia elongation, associated with a failure of p190A_L1396Q_ to be recruited to the base of the cilium. *(A)* Immunofluorescent staining of MEFs stimulated to form cilia by serum withdrawal reveals a defect in cilia length (acetylated α-tubulin, open arrowhead) independent of the basal body (γ-tubulin, closed arrowhead). *(B)* Quantification of number of ciliated cells based on basal body (γ-tubulin) and axoneme (acetylated α-tubulin). *(C)* The positioning of the basal body with respect to the cell surface is similar between control and *Arhgap35*^*D34/D34*^ MEFs. *(D-E)* The defects in cilia number (panel D) and length (panel E) in *Arhgap35*^*D34/D34*^ MEFs can be rescued by introduction of full-length wild-type p190A, but not the p190A_L1396Q_ mutant protein. *(F)* Full-length GFP-tagged p190A_WT_ and p190A_GAP_ constructs are enriched at the basal body (marked by γ-tubulin; arrowheads) while p190A_L1396Q_, P190A_R1284A_, p190A_GBD_, and p190A_GBD_FF_ constructs fail to specifically localize in wild type MEFs. The percentage of cells with ciliary localization is indicated (N>200 cells per construct), which suggests a transient recruitment to the basal body. Scale bars, 2.5μm. *p<0.05, **p<0.01, ***p<0.005 (one-way ANOVA)

To understand the mechanistic relationship between p190A and cilium formation, we turned to the more tractable system of primary mouse embryonic fibroblasts (MEFs), which reproducibly form cilia upon serum withdrawal. Whereas approximately 60% of wild type MEFs formed cilia, less than 20% of *Arhgap35*^*D34/D34*^ MEFs produced elongated cilia and the cilia that did form were on average half the length of wild type (Figs 5A, 5E and S7A). Additionally, expression of a GFP-p190A_WT_ full length construct, but not GFP-p190A_L1396Q_, in *Arhgap35* mutant MEFs restored their ability to form elongated cilia to wild-type parameters (Fig 5D and 5E), further confirming that the cilium defect results from a deficiency in p190A activity. In line with the phenotype observed in proximal tubule cells, immunofluorescent staining of control and mutant MEFs with γ-tubulin showed a normal number of basal body-containing cells, which were docked appropriately at the plasma membrane (Fig 5A, 5B and 5C).

We next examined whether p190A was present at the cilium. Introduction of the GFP-p190A_WT_ construct in wild type MEFs revealed an enrichment of p190A co-localizing with γ-tubulin at the base of cilia in 12% of cells (Fig 5F), consistent with a direct, and transient role for p190A in cilia formation. Interestingly, this enrichment was not observed in MEFs expressing the D34-mutant form of p190A (GFP-p190A_L1396Q_) (Fig 5F), suggesting that a functional RhoGAP domain is necessary for p190A recruitment to basal bodies. In line with this result, p190A was also absent from the basal body in MEFs expressing a catalytically dead p190A variant (GFP-p190A_R1284A_) (Fig 5F). We further generated deletion constructs fused to GFP, which identified the C-terminal GAP domain as sufficient for basal body localization (Fig 5F). Together, these results demonstrate the causal role of the p190A_L1396Q_ mutation in the cilia phenotype of *Arhgap35*-deficient cells.

### P190A regulates actin cytoskeletal dynamics to permit cilia elongation

**Fig 6:**
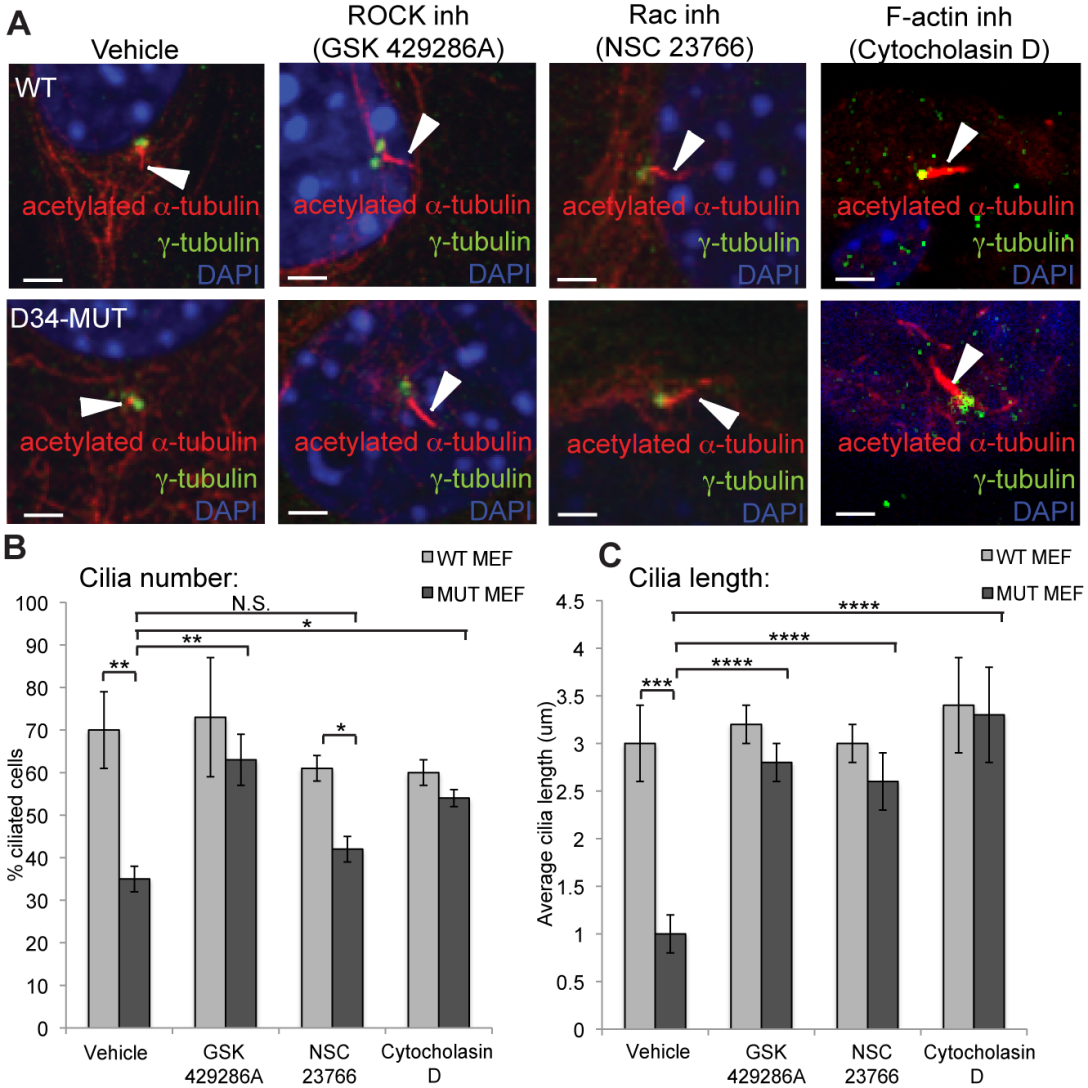
Ciliogenesis defects in *Arhgap35*^*D34/D34*^ MEFs can be rescued by inhibition of Rho GTPases and depolymerization of F-actin. *(A-C)* Wild type and *Arhgap35*^*D34/D34*^ MEFs were stimulated to form cilia by serum withdrawal and treated with inhibitors of ROCK1/2 (GSK 429286A), Rac1 (NSC 23766) or the F-actin polymerization inhibitor Cytocholasin D. *(A)* Ciliary defects of *Arhgap35*^*D34/D34*^ MEFs are fully rescued by ROCK1/2 and F-actin inhibition and partially rescued by Rac1 inhibition as shown by immunofluorescence staining against γ-tubulin (basal body) and acetylated α-tubulin (axoneme)(arrowheads). Scale bars, 2.5μm *(B-C)* Quantification of cilia number (B) and length (C) in wild type and mutant MEFs treated with ROCK, Rac and F-actin polymerization inhibitors from (A). *p<0.05, **p<0.01, ***p<0.005, ****p<0.001 (one-way ANOVA)

Recently, the impact of the actin cytoskeleton on ciliogenesis has been garnering attention, notably on the balance between free versus filamentous actin in promoting cilia elongation [38,39,40,41,42,43,52]. Given that the p190A-L1396Q mutation results in a loss of GAP activity toward RhoA and Rac1 (Figs 3C, 3D and S3D), we predicted that their downstream pathways would be activated, potentially perturbing actin cytoskeletal dynamics. To evaluate the relative contribution of Rho and Rac signaling to ciliogenesis in the presence or absence of p190A function, we used small molecule inhibitors of Rho-kinases 1/2 (ROCK1/2) and Rac1. Remarkably, at concentrations that do not alter the onset of ciliogenesis in control MEFs, inhibition of ROCK1/2, using either Y27632 [53] or GSK 429286A [54], completely restored cilia numbers and cilia length of *Arhgap35*^*D34/D34*^ MEFs to wild-type levels (Figs 6, S7B and S7C). In contrast, blocking Rac1 activation using NSC23766 [55] did not have a significant effect on overall cilia numbers but allowed for better elongation in forming cilia (Fig 6).

**Fig 7:**
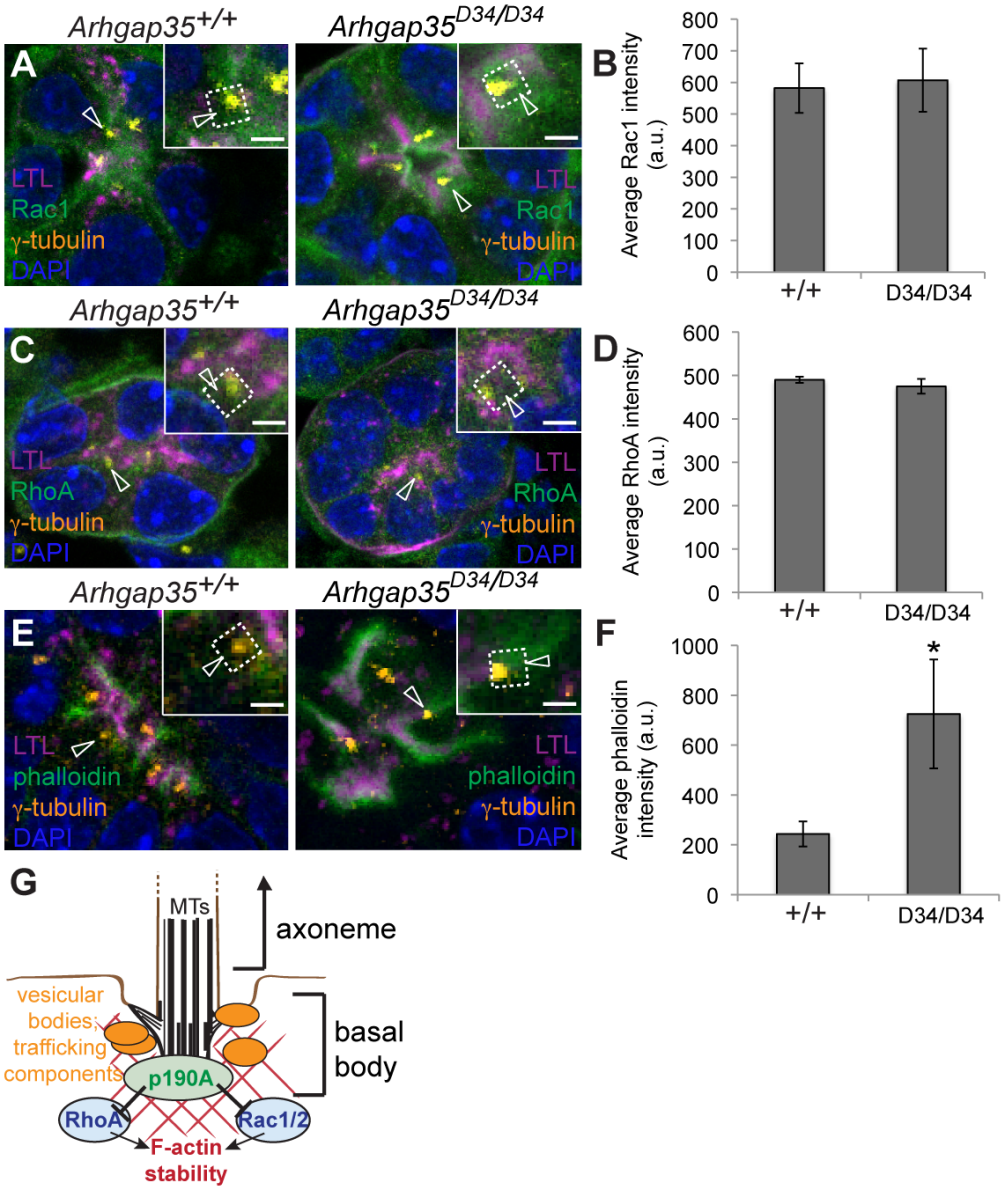
Ciliogenesis defects in *Arhgap35*^*D34/D34*^ proximal tubules are associated with excessive RhoGTPase-dependent F-actin polymerization. *(A*,*C*,*E)* Immunofluorescent staining for Rac1 (*A*), RhoA (*C*), and phalloidin (*E*) in E17.5 wild type and *Arhgap35*^*D34/D34*^ proximal tubules (marked by Lotus Tetragonolobus Lectin; LTL) reveals an increase in downstream F-actin (phalloidin) intensity around the basal body (γ-tubulin) in *Arhgap35*^*D34/D34*^ animals, despite normal levels of Rac1 and RhoA. Scale bars, 2μm *(B*,*D*,*F)* Quantification of staining intensity from panels A,C,E within 2μm of the basal body (N = 90 basal bodies from 3 animals for each genotype). *p<0.05 (unpaired, two-tailed Student’s *t*-test)

The complete rescue of ciliogenesis by ROCK1/2 inhibition pointed to an important role for RhoA signalling in the *Arhgap35*-deficient phenotype. Surprisingly, however, phalloidin-labelled F-actin formed normal cytoskeleton networks in both gain and loss of function analyses of p190A function (S7H Fig), suggesting that aberrations in ciliogenesis do not result from global alterations in stress fibre formation. Accordingly, inhibition of myosin light chain II activity by Blebbistatin [56] failed to rescue both the number and length of cilia in *Arhgap35*^*D34/D34*^ MEFs, indicating that in this system RhoA-Rock1/2 ectopic activation does not act through the actomyosin pathway (S7D and S7E Fig). To validate that the ciliogenesis defect was nonetheless a result of excess F-actin formation, we quantified cilium number and length in the presence of Cytochalasin D or Latrunculin A, two small molecules that prevent F-actin polymerization [41,57]. Strikingly, Cytochalasin D or Latrunculin A treatment restored both cilium number and length of *Arhgap35*^*D34/D34*^ MEFs to wild type levels (Figs 6, S7F and S7G), suggesting that p190A may normally dampen Rho and Rac-dependent F-actin polymerization to permit cilia elongation. To determine whether reduced local actin polymerization also underlies the ciliogenesis defect observed in vivo, we examined the localization of Rac1, RhoA and F-actin in proximal tubules of control and *Arhgap35*^*D34/D34*^ kidneys by immunofluorescent staining. In both wild type and *Arhgap35*^*D34/D34*^ proximal tubules Rac1 and RhoA are enriched at the basal bodies to similar degrees (Fig 7A–7D). In contrast, *Arhgap35*^*D34/D34*^ animals exhibited a marked increase in the amount of F-actin polymerized around the basal body compared to control animals (Fig 7E and 7F). Together, these results point to a permissive role for p190A specifically at the basal body to promote ciliogenesis through a local dampening of Rho GTPase-dependent F-actin polymerization (Fig 7G).

## Discussion

Using an ENU mutagenesis approach, we have identified a new allele of the p190A RhoGAP gene *Arhgap35*, which leads to defects in renal morphogenesis. In particular, we found that *Arhgap35* mutation results in hypoplastic kidneys with a fully penetrant glomerulocystic phenotype associated with defective cilia elongation. Together these results establish a strong and unexpected link between the modulation of Rho family GTPases by p190A RhoGAP, ciliogenesis and renal developmental diseases.

Using a combination of classical single nucleotide polymorphism (SNP) mapping and exome sequencing approaches, we have identified a point mutation in *Arhgap35*. The causal link between this sequence variant and the developmental phenotype was demonstrated both by genetic non-complementation with an independent *Arhgap35* mutant strain [16], and by rescue of the ciliary defect in *Arhgap35*-deficient MEFs by reintroduction of wild type but not mutant p190A. The similar penetrance of neural tube and renal defects between *Arhgap35*^*D34*^ and the previous reports on *Arhgap35* mutant alleles [16,18] identifies D34 as a null or a strong hypomorph allele. In line with this, functional testing of the p190A_L1396Q_ variant in vitro led to a significant loss of activity on the prototypical Rho family GTPases. Based on the structural and mechanistic similarity between Rho family GTPases and their cognate GAPs, three critical residues (R1284, K1322, N1395) have been identified that together define a shallow Rho-binding pocket on p190A comprised of two α-helices and the catalytic arginine loop [8,48,49]. Interestingly, the hydrophobic L1396 residue is buried between these helices. Modeling the mutant glutamine reveals steric clashes with the surrounding amino acids and likely results in disruption of this α-helical interface, and subsequent distortion of the Rho-binding surface. Furthermore, GTPase activity requires stabilization of the catalytic loop by N1395 to optimally position GTP for hydrolytic attack in the transition state intermediate [8,49]. It seems likely that substitution of the longer and hydrophilic glutamine side chain at position 1396, adjacent to N1395, affects this critical interaction point, thus lowering the efficiency of catalytic loop stabilization and resulting in the markedly reduced activity observed in vitro.

Unexpectedly, we found that the reduction in p190A GAP activity perturbs cilia elongation in both kidney proximal tubule cells and mouse embryonic fibroblasts, associated with dysregulated Rho GTPase signaling. The pharmacological manipulations of *Arhgap35*-deficient cells suggest that both Rac1/2 and RhoA may be substrates for p190A during cilia formation; however, while Rac inhibition only partially rescues ciliogenesis in the absence of p190A activity, ROCK inhibition completely rescues both cilia number and length. Together, these results raise the intriguing possibility that p190A plays a role at the docked basal body to downregulate active RhoA (and possibly Rac1) to allow the localized actin cytoskeleton rearrangement required for cilia elongation (Fig 7G). In support of this, full-length p190A and the RhoGAP domain alone are enriched at the base of the cilia, while p190A_L1396Q_ and p190A_R1284A_ that lacks RhoGAP activity fails to be recruited. Additionally, we find that the F-actin inhibitory drugs Cytochalasin D and Latrunculin A are able to rescue the defects in cilia number and length seen in the absence of functional p190A. Furthermore, as predicted, we observe increased F-actin around the basal bodies of *Arhgap35*^*D34/D34*^ proximal tubules. These results confirm that p190A deficiency results in ectopic actin polymerization, leading to compromised cilia elongation. This finding is in line with other studies of ciliogenesis which identified F-actin modifying proteins, including regulators of actin branch nucleation and filament stability, that restrict primary cilia elongation [38,39,40,41,42,43,52,58]. Notably, however, the upstream Rho GTPases involved remained unclear. Our study provides the first genetic demonstration that the negative regulation of Rho GTPases at the basal body is required for primary cilium elongation in multiple cell types.

Interestingly, cilia have been observed on the ureteric bud epithelium during branching morphogenesis, raising the possibility that earlier cilia defects may also be causative in the renal hypoplasia observed. Cilia elongation is also impaired in early nephrogenic structures upon loss of p190A activity; however, the functional relevance of this observation awaits future studies.

Together our results establish a link between Rho GTPase regulation, ciliogenesis and glomerular cystic defects. It provides a molecular and cell biological basis for understanding glomerulocystic kidney disease, a form of renal cystic disease that is less frequent and less well understood than polycystic kidney disease that primarily affects tubular nephrons and collecting ducts [59]. The finding that the ciliogenesis defect of *Arhgap35*-deficient kidneys primarily affects proximal tubule cells, which correlates with the site of high *Arhgap35* transcriptional expression, raises the interesting possibility that glomerulocystic kidney disease can, in some instances, be secondary to proximal tubule deficiencies. This work additionally identifies Rho GTPases and their associated GAPs, and GEFs, as candidates in the etiology of glomerulocystic kidney disease.

## Materials and Methods

### Ethics statement

Experiments for this study were approved by the McGill Animal Care Committee, and were conducted in compliance with the Canadian Council of Animal Care ethical guidelines for animal experiments.

### ENU mutagenesis screen

N-ethyl-N-nitrosourea (ENU, 90 mg/Kg) was administered as 3 weekly doses by intraperitoneal injections to 8-week old male C57BL/6J mice (Jackson Laboratories) to induce germline mutations as described previously [60]. G0 founder males were outcrossed to wild type C3H/HeNrl females (Charles River Laboratories) to establish 30 G1 lines for screening. Phenotypes in the D34 line were analyzed between generations four and twelve, with establishment of two independent *Arhgap35*^*D34*^ lines at generation seven by breeding *Arhgap35*^*+/D34*^ males wild type females. Kidneys were identified as hypoplastic if they were <75% of littermate size. Restriction fragment length polymorphism (RFLP) analysis was used to genotype animals by PCR-amplifying 600bp fragments around the single nucleotide polymorphisms (SNPs) rs31200925 and rs31924991. Mice carrying the C3H/HeNCrl (wild-type) allele at these SNPs have restriction sites for Bgl II and Hind III, respectively, while animals with the C57BL/6J (mutated) allele do not. Restriction digest of the PCR product results in fragments of 600bp (C57BL/6J) and/or 400bp and 200bp (C3H/HeNCrl) allowing for genotype determination. When appropriate, *Arhgap35* was also PCR-amplified for Sanger sequencing. All primers used are provided in S1 Table.

### Mutation identification

SNP Array screening was performed on the Low-density Mouse Linkage Panel (LD panel, Golden Gate technology, Illumina). R-QTL mapping was performed using the R software platform [61]. Whole Exome sequencing was performed with Illumina Nextera Exome capture technology on 5 affected samples, 1 littermate control, 1 wild type C3H/HeNrl and 1 C57BL/6J sample. SNP Array and exome sequencing experiments were conducted by the McGill University and Genome Quebec Innovation Centre (Montreal, Canada).

### P190A expression constructs

Full length p190A-pEGFP-C1 mammalian expression vector was generated as described [10]. The GFP-p190A_L1396Q_ and GFP-p190A_R1284A_ constructs were generated from the full-length wild-type construct by site directed mutagenesis. For the GFP-p190A domain constructs, the appropriate domains were PCR amplified from the full-length wild-type construct. For in vitro GAP assays, the GAP domain (nt 4417–5143) was PCR amplified from the either the full length GFP-p190A_WT_ or GFP-p190A_L1396Q_ constructs using specific primers containing BglII and EcoRI restriction sites, which allowed for cloning in the BamHI and EcoRI sites of the pGEX-6P1 bacterial expression vector. All constructs were sequence verified. All primers used are provided in S1 Table.

### In vitro p190A GAP assay

Recombinant GST-tagged p190A GAP protein was produced in BL21(DE3) bacteria by conventional methods, and purified using Glutathione-agarose beads (Life Technologies). Cleavage of the GST tag, and elution of free p190A GAP protein, was achieved using PreScission protease (GenScript). Protein was quantified so that 1.5ug of GAP was added per GAP assay reaction. The in vitro GAP assay (Cytoskeleton) was performed according to manufacturer’s instructions.

### In vivo active RhoA assay

The amount of activated RhoA (RhoA-GTP) was assessed using the Rho Activation Assay Biochem Kit (Cytoskeleton) following manufacturer’s instructions. Briefly, control and mutant MEFs were grown to 70% confluence, and serum starved for 16hr to assess the basal level of RhoA activation. Cells were lysed on ice, clarified by centrifugation and snap frozen. 400ug of each lysate was used for pulldown assay using the Rho binding domain from rhotekin conjugated to sepharose beads. The amount of activated RhoA was assessed by western blot, following standard procedures. The primary antibodies used were mouse anti-RhoA (1:500, Cytoskeleton) and rabbit anti-GAPDH (1:5000, Abgent). Secondary antibodies used were HRP-conjugated anti-mouse (1:10000, GE Healthcare) and HRP-conjugated anti-rabbit (1:10000, Cell Signaling Technology).

### Structural analysis

To visualize the potential impact of the L1396Q substitution on the structure of p190A RhoGAP, the Pymol software program was used to image the human p190A GAP domain (PDB: 3FK2). Substitution of glutamine for leucine 1396 was done using the mutagenesis function of Pymol, and the rotamer position of the glutamine side chain and potential steric clashes with surrounding amino acids were assessed for all possible conformations.

### Histology, immunofluorescence and in situ hybridization

Dissected urogenital systems (UGS) were formalin-fixed, paraffin embedded and processed according to standard methods for Hematoxylin and Eosin staining at the McGill University Histology Facility. Section immunofluorescence and in situ hybridizations were performed on dissected UGS, fixed in 4% paraformaldehyde and cryo-sectioned as described previously [62]. Probes for *Gata3*, *Ncc*, *and Wt1* have been described [63,64,65]. The *Arhgap35* probe was generated from E12.5 whole embryo cDNA using specific primers containing a T7 promoter sequence (S1 Table). Probe was transcribed following standard procedures using T7 RNA polymerase and DIG-labeled dNTPs (Roche). For immunohistochemical stainings, sections were permeabilized with 0.3% Triton X-100 in PBS for 10 minutes, blocked with DAKO protein block (serum-free), and incubated with the following primary antibodies or Lectin overnight at 4°C: rabbit anti-laminin (1:200, Sigma), biotinylated-Lotus Tetragonolobus Lectin (1:1000, Vector Laboratories), guinea pig anti-nephrin (1:200, Acris Antibodies Inc.), mouse anti-synaptopodin (1:200, Progen Biotechnik), mouse anti-acetylated tubulin (1:1000, Sigma), mouse anti-Arl13b (1:100, NeuroMab), goat anti-γ tubulin (1:100, Santa Cruz Biotechnology Inc.), mouse anti-Wt1 (1:100, DAKO), and rat anti-ZO-1+ (1:200, Chemicon). Secondary detection was performed using donkey anti-rabbit, anti-mouse, anti-guinea pig or anti-goat secondary antibodies labeled with AlexaFluor-488, -568, or -647 (1:400, Invitrogen) or FITC-conjugated streptavidin (1:1000, Zymed), as well as DAPI (50ug/ml, Invitrogen) and mounted with SlowFade Gold (Invitrogen). For F-actin visualization AlexaFluor-568 or -635 conjugated phalloidin (1:400, Invitrogen) was added with the secondary antibodies. For all stainings, at least 3 animals of each genotype were examined.

### Cell culture

Mouse embryonic fibroblasts were derived as described previously [62] from *Arhgap35*^*+/+*^ and *Arhgap35*^*D34/D34*^ embryos. Cells were maintained in DMEM (Wisent Inc.) supplemented with 10% FBS (Gibco), 1% Penicillin-Streptomyosin (Wisent Inc.). Ciliogenesis was stimulated by serum withdrawal for 20 hours before addition of Y27632 (0.5μM and 1μM, Sigma), GSK 429286A (15nM, Selleck Chemicals), or NSC 23766 (25μM, Tocris Bioscience) for an additional 4 hours. Alternatively, for experiments involving Blebbistatin (10μM, Sigma), Latrunculin A (1μM, Sigma) or Cytochalasin D (0.5μM, Calbiochem), ciliogenesis was stimulated for 23.5 hours prior to treating with the inhibitor for an additional 30 minutes. Cells were then fixed with 4% paraformaldehyde, and processed for immunofluorescence. P190A localization during ciliogenesis was examined by transfecting the indicated full length GFP-p190A constructs in mouse embryonic fibroblasts (MEFs) using the Nucleofector technology (Lonza). Twenty-four hours post transfection, ciliogenesis was stimulated by serum withdrawal for an additional 24 hours. Cells were then fixed with 4% paraformaldehyde and processed for immunofluorescence. A minimum of 50 cells were assessed in each sample.

### Imaging and statistical analysis

Images were acquired on a Confocor LSM _510_ META Axiovert 200M inverted microscope at the Advanced BioImaging Facility of McGill University (Montreal, Canada). Cilia length and basal body positioning were determined using the measure function of the ImageJ image analysis program [66] from confocal projections. Statistical analyses were performed as either one-way ANOVA or unpaired, two-tailed Student’s *t*-tests (as indicated) using GraphPad Prism 6 software. Data are presented as mean ± SEM; p<0.05 was considered significant.

## Supporting Information

S1 Fig Breeding scheme of a recessive ENU mutagenesis screen. N-ethyl-N-nitrosurea (ENU)-mutagenized male C57BL/6J mice were outcrossed to wild-type C3H/HeNCrl females to produce first generation (G1) offspring. Sons from this cross were outbred to wild-type C3H/HeNCrl females to produce the second generation (G2), from which daughters were backcrossed to their father to recover recessive mutations in the third generation (G3). G3 progeny were dissected at embryonic day 17.5 (E17.5) for gross characterization that identified the D34 line.
(TIF)

S2 Fig In situ hybridization and immunohistochemical marker analysis shows normal nephrogenesis in *Arhgap35*^*D34/D34*^ kidneys at E17.5. *(A-B)* Section in situ hybridization on control and D34-mutant kidneys shows no significant difference in collecting ducts and ureteric tips marked by *Gata3* expression (A), nor in distal tubule density and differentiation marked by *Ncc* (B) expression. *(C)* Section immunohistochemistry for Lotus Tetragonolobus Lectin (LTL) on control and *Arhgap35*^*D34/D34*^ kidneys shows no difference in proximal tubule formation. *(D) Arhgap35*^*D34/D34*^ kidneys contain significant increases in glomerular and proximal tubule cysts compared to wild-type E17.5 kidneys. Scale bars, 100μm *p<0.05, **p<0.01, ***p<0.005 (unpaired, two-tailed Student’s *t*-test)
(TIF)

S3 Fig Neural tube closure defects and glomerulocystic kidneys are present at similar penetrance in *Arhgap35*^*D34/D34*^, *Arhgap35*^*-/-*^, and *Arhgap35*^*-/D34*^ embryos. *(A-C)* Whole E17.5 embryos that are *Arhgap35*^*-/-*^ or compound heterozygous with the *Arhgap35*^*D34*^ allele exhibit spina bifida (closed arrowhead) and exencephaly (open arrowhead). *(D)* Active RhoA in mouse embryonic fibroblasts derived from wild type or *Arhgap35D*^*34/D34*^ animals was assessed by pulldown with Rho binding domain (RBD) bound beads. GAPDH was used for normalization. *(E)* Denisometric analysis of (D) was performed using ImageJ to reveal an increase in active RhoA in D34-mutant cells.
(TIF)

S4 Fig Glomerular morphogenesis is initially normal in *Arhgap35*^*D34/D34*^ embryos. *(A)* In situ hybridization of E17.5 kidney section for *Wt1* shows a normal progression of podocyte development. *(B)* Quantification of the average density of H&E-stained glomeruli normalized to kidney area shows no difference between control and *Arhgap35*^*D34/D34*^ animals (unpaired, two-tailed Student’s *t*-test). *(C-D)* Immunofluorescence staining for podocyte markers (Wt1, Synaptopodin) and structural markers (phalloidin, laminin) show no obvious misorganization in *Arhgap35*^*D34/D34*^ glomeruli, either pre- or post-cyst formation (arrowheads). *(E)* Section immunofluorescence for cilia (acetylated α-tubulin) and the podocyte lineage (podocalyxin) shows normal cilia in Bowman’s capsule cells (visualized with DAPI). *(F)* Quantification of cilia number per glomerulus from *(F)* shows no significant difference between control and *Arhgap35*^*D34/D34*^ animals, irrespective of glomerular dilation (unpaired, two-tailed Student’s *t*-test). Scale bars, 10μm
(TIF)

S5 Fig *Arhgap35* is expressed during kidney development. *(A*,*B)* Section in situ hybridization (20X magnification) for *Arhgap35* on wild type and D34-mutant animals shows weak expression in distal tubules (DT), collecting ducts (CD), and nephrogenic zone. *(C*,*D)* Section in situ hybridization (63X magnification) for *Arhgap35* on wild type and *Arhgap35*^*D34/D34*^ embryos shows strong expression in proximal tubules (PT) but only weak expression in the glomerulus (G). Scale bars, 50μm
(TIF)

S6 Fig Defective ciliogenesis precedes the onset of cystic disease. *(A)* Immunofluorescence staining for γ-tubulin (basal body) and acetylated α-tubulin (axoneme) reveal a defect in cilia elongation (closed arrowheads) in the S-shaped body (dotted lines) of *Arhgap35*^*D34/D34*^ animals. Scale bars, 10μm *(B)* Immunofluorescence staining for acetylated α-tubulin (axoneme) of E14.5 proximal tubules (marked by Lotus Tetragonolobus Lectin, LTL) shows a defect in cilia elongation (open arrowheads) in *Arhgap35*^*D34/D34*^ animals that precedes cystogenesis. Scale bars, 5μm
(TIF)

S7 Fig Cilia elongation is disrupted in *Arhgap35*^*D34/D34*^ cells, independent of actomyosin fibre formation. *(A)* Immunofluorescence staining for γ-tubulin (basal body) and Arl13b (axoneme) reveal a defect in cilia elongation in *Arhgap35*^*D34/D34*^ mouse embryonic fibroblasts compared to control. *(B-C)* Treatment with the ROCK1/2 inhibitor, Y27632, rescues the defects in cilia number (B) and cilia length (C) in *Arhgap35*^*D34/D34*^ mouse embryonic fibroblasts. *(D)* Immunofluorescence for phalloidin and acetylated α-tubulin in control and *Arhgap35*^*D34/D34*^ mouse embryonic fibroblasts shows a normal F-actin cytoskeleton in ciliated cells. Staining for phalloidin in mouse embryonic fibroblasts overexpressing full length p190A_WT_-GFP shows no noticeable effect on F-actin organization. *(E-F)* Treatment with the myosin II inhibitor Blebbistatin does not rescue the defects in cilia number (D) and cilia length (E) in *Arhgap35*^*D34/D34*^ mouse embryonic fibroblasts. *(G-H)* Treatment with the actin polymerization inhibitor Latrunculin A rescues the defects in in cilia number (G) and cilia length (H) in *Arhgap35*^*D34/D34*^ mouse embryonic fibroblasts. *p<0.05, **p<0.01, ***p<0.005, ****p<0.001 (one-way ANOVA)
(TIF)

S1 Table Polymerase chain reaction primers used for genotyping and cloning of *Arhgap35*. (XLSX)
