# Supplementary figures and images for: A Point Mutation in p190A RhoGAP Affects Ciliogenesis and Leads to Glomerulocystic Kidney Defects
Source: PLoS Genet. 2016 Feb 9;12(2):e1005785. doi: 10.1371/journal.pgen.1005785 (PMC4747337; doi:10.1371/journal.pgen.1005785)

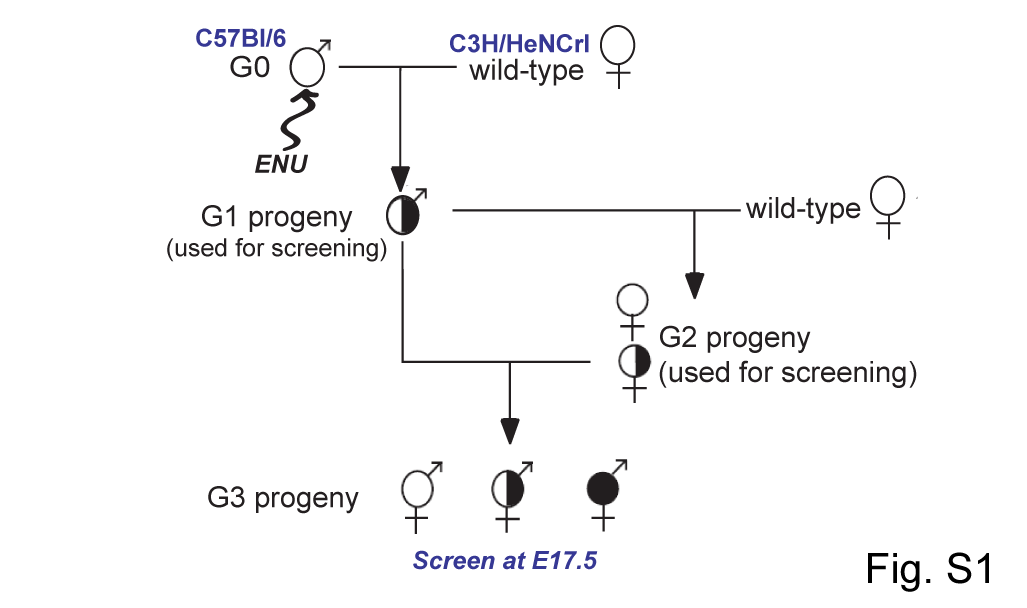

Supplement: S1 Fig — N-ethyl-N-nitrosurea (ENU)-mutagenized male C57BL/6J mice were outcrossed to wild-type C3H/HeNCrl females to produce first generation (G1) offspring. Sons from this cross were outbred to wild-type C3H/HeNCrl females to produce the second generation (G2), from which daughters were backcrossed to their father to recover recessive mutations in the third generation (G3). G3 progeny were dissected at embryonic day 17.5 (E17.5) for gross characterization that identified the D34 line. (TIF) [file pgen.1005785.s001.tif]

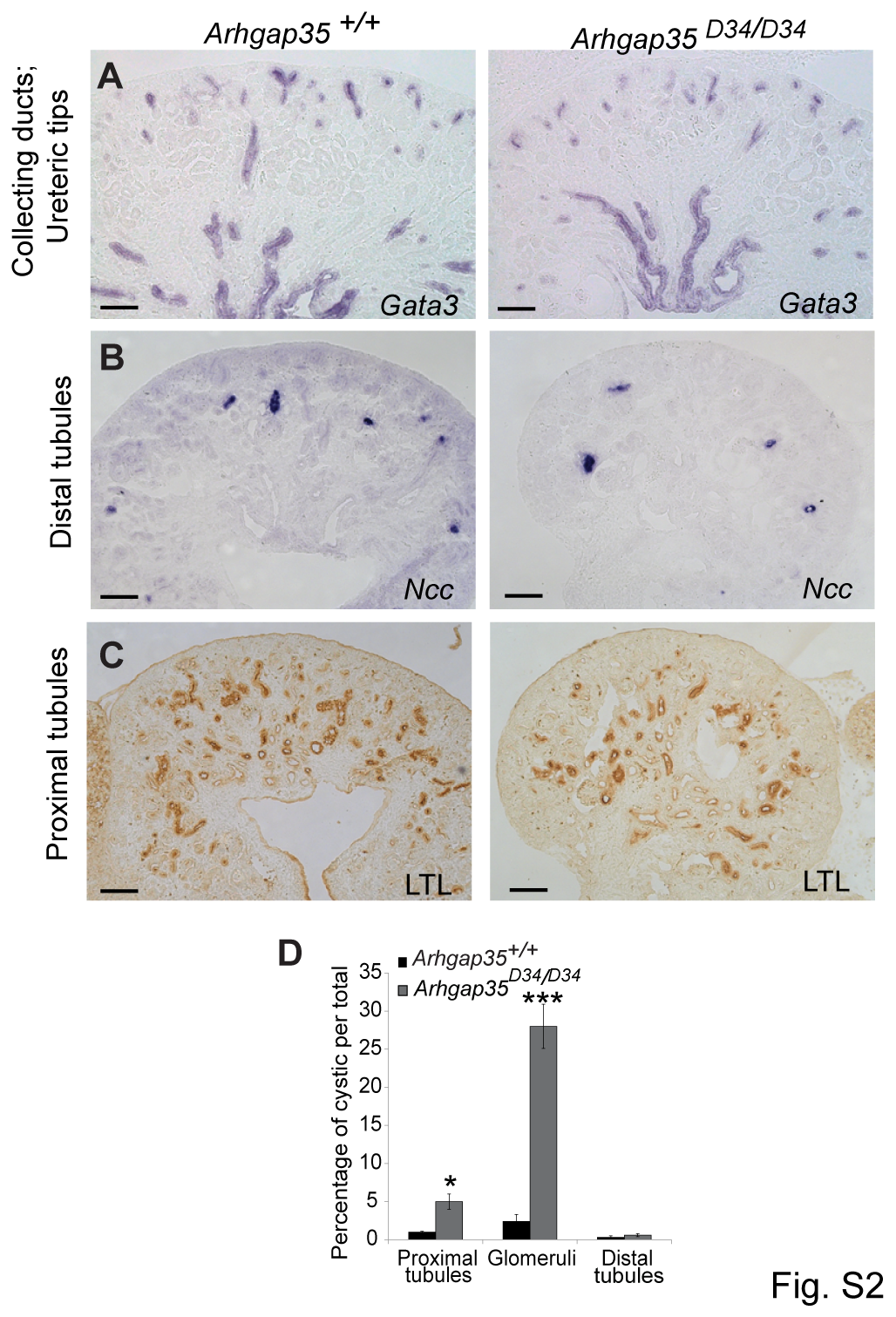

Supplement: S2 Fig — (A-B) Section in situ hybridization on control and D34-mutant kidneys shows no significant difference in collecting ducts and ureteric tips marked by Gata3 expression (A), nor in distal tubule density and differentiation marked by Ncc (B) expression. (C) Section immunohistochemistry for Lotus Tetragonolobus Lectin (LTL) on control and Arhgap35D34/D34 kidneys shows no difference in proximal tubule formation. (D) Arhgap35D34/D34 kidneys contain significant increases in glomerular and proximal tubule cysts compared to wild-type E17.5 kidneys. Scale bars, 100μm *p<0.05, **p<0.01, ***p<0.005 (unpaired, two-tailed Student’s t-test) (TIF) [file pgen.1005785.s002.tif]

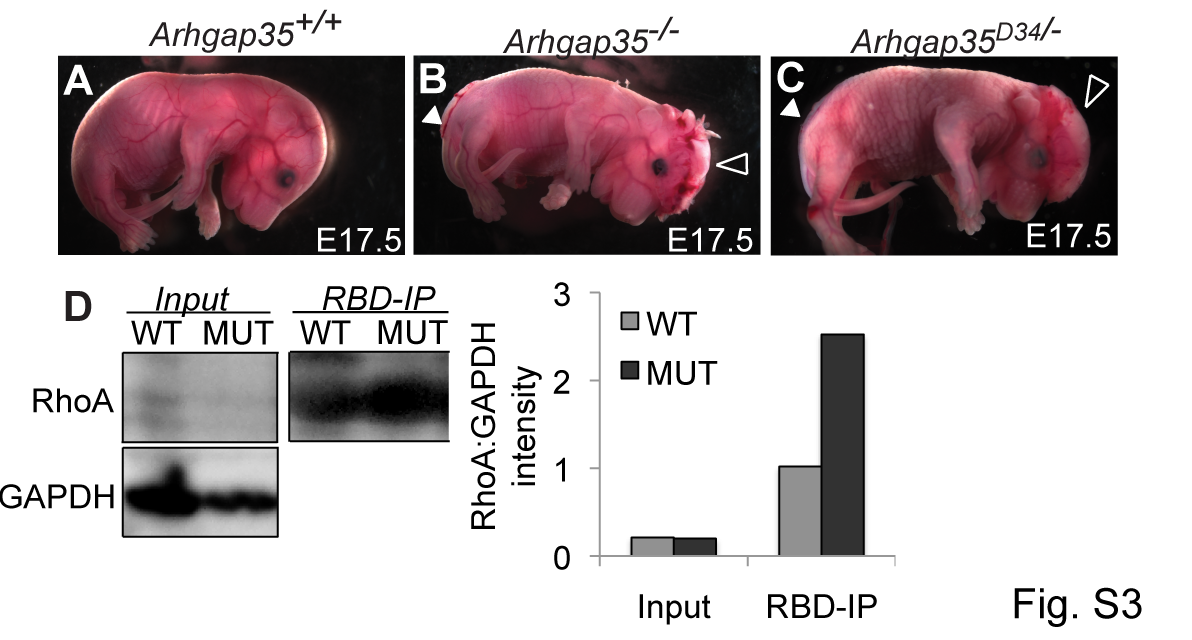

Supplement: S3 Fig — (A-C) Whole E17.5 embryos that are Arhgap35-/- or compound heterozygous with the Arhgap35D34 allele exhibit spina bifida (closed arrowhead) and exencephaly (open arrowhead). (D) Active RhoA in mouse embryonic fibroblasts derived from wild type or Arhgap35D34/D34 animals was assessed by pulldown with Rho binding domain (RBD) bound beads. GAPDH was used for normalization. (E) Denisometric analysis of (D) was performed using ImageJ to reveal an increase in active RhoA in D34-mutant cells. (TIF) [file pgen.1005785.s003.tif]

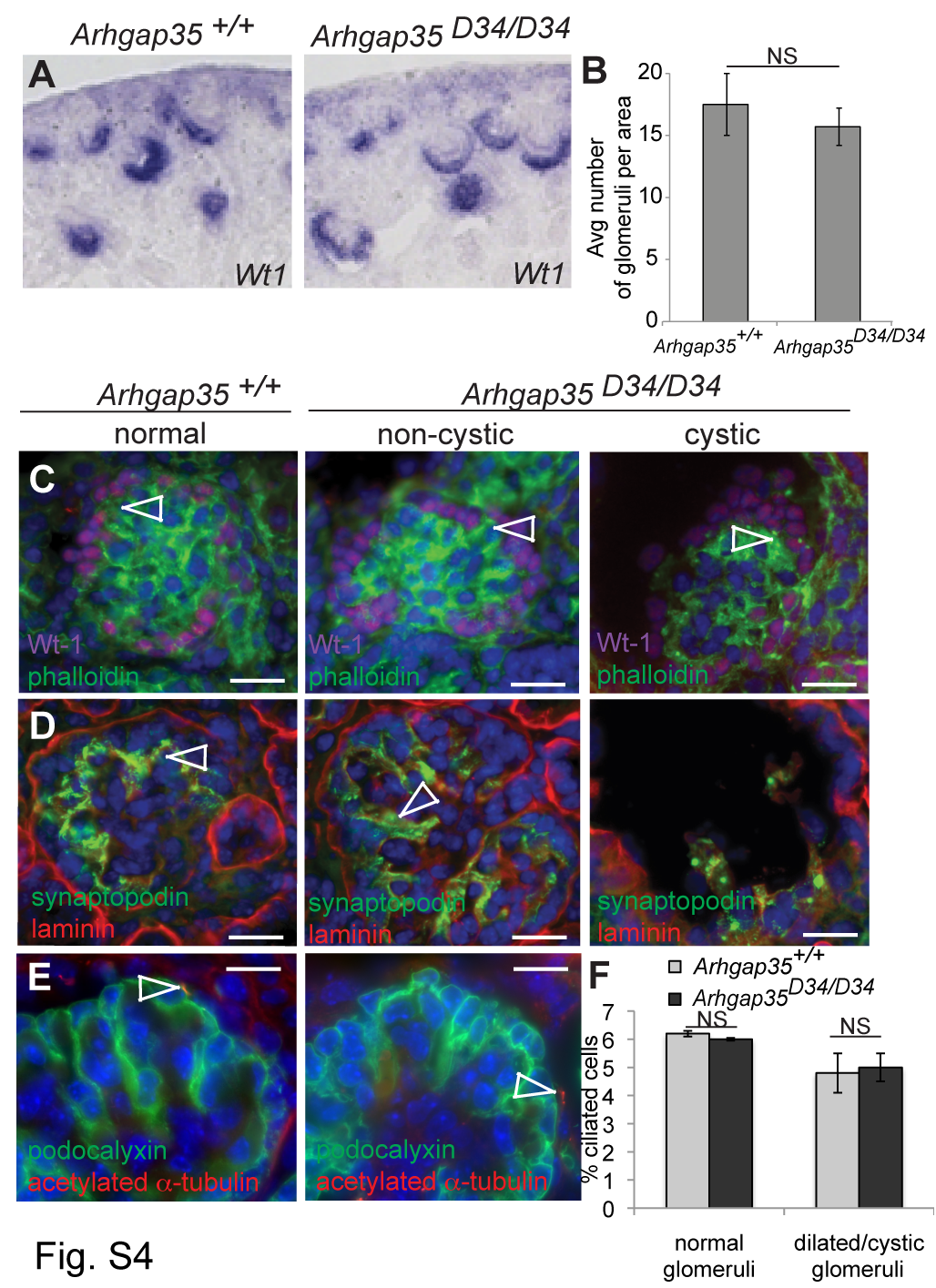

Supplement: S4 Fig — (A) In situ hybridization of E17.5 kidney section for Wt1 shows a normal progression of podocyte development. (B) Quantification of the average density of H&E-stained glomeruli normalized to kidney area shows no difference between control and Arhgap35D34/D34 animals (unpaired, two-tailed Student’s t-test). (C-D) Immunofluorescence staining for podocyte markers (Wt1, Synaptopodin) and structural markers (phalloidin, laminin) show no obvious misorganization in Arhgap35D34/D34 glomeruli, either pre- or post-cyst formation (arrowheads). (E) Section immunofluorescence for cilia (acetylated α-tubulin) and the podocyte lineage (podocalyxin) shows normal cilia in Bowman’s capsule cells (visualized with DAPI). (F) Quantification of cilia number per glomerulus from (F) shows no significant difference between control and Arhgap35D34/D34 animals, irrespective of glomerular dilation (unpaired, two-tailed Student’s t-test). Scale bars, 10μm (TIF) [file pgen.1005785.s004.tif]

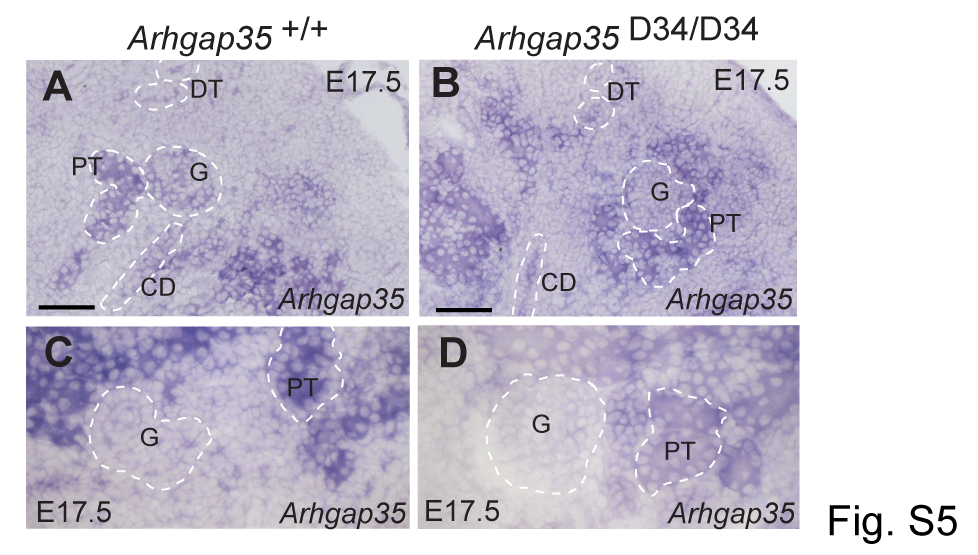

Supplement: S5 Fig — (A,B) Section in situ hybridization (20X magnification) for Arhgap35 on wild type and D34-mutant animals shows weak expression in distal tubules (DT), collecting ducts (CD), and nephrogenic zone. (C,D) Section in situ hybridization (63X magnification) for Arhgap35 on wild type and Arhgap35D34/D34 embryos shows strong expression in proximal tubules (PT) but only weak expression in the glomerulus (G). Scale bars, 50μm (TIF) [file pgen.1005785.s005.tif]

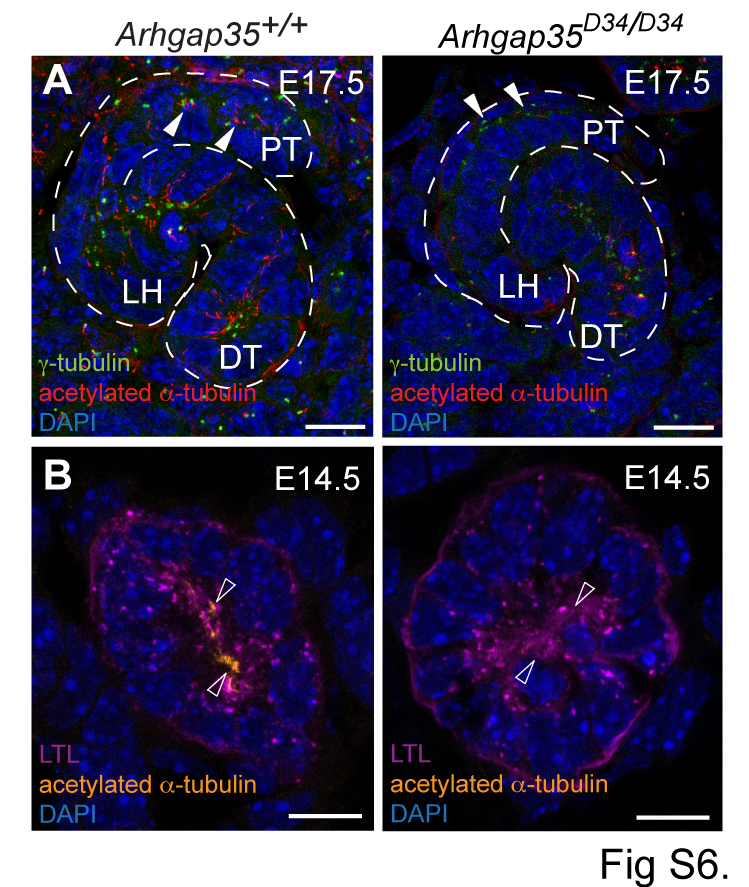

Supplement: S6 Fig — (A) Immunofluorescence staining for γ-tubulin (basal body) and acetylated α-tubulin (axoneme) reveal a defect in cilia elongation (closed arrowheads) in the S-shaped body (dotted lines) of Arhgap35D34/D34 animals. Scale bars, 10μm (B) Immunofluorescence staining for acetylated α-tubulin (axoneme) of E14.5 proximal tubules (marked by Lotus Tetragonolobus Lectin, LTL) shows a defect in cilia elongation (open arrowheads) in Arhgap35D34/D34 animals that precedes cystogenesis. Scale bars, 5μm (TIF) [file pgen.1005785.s006.tif]

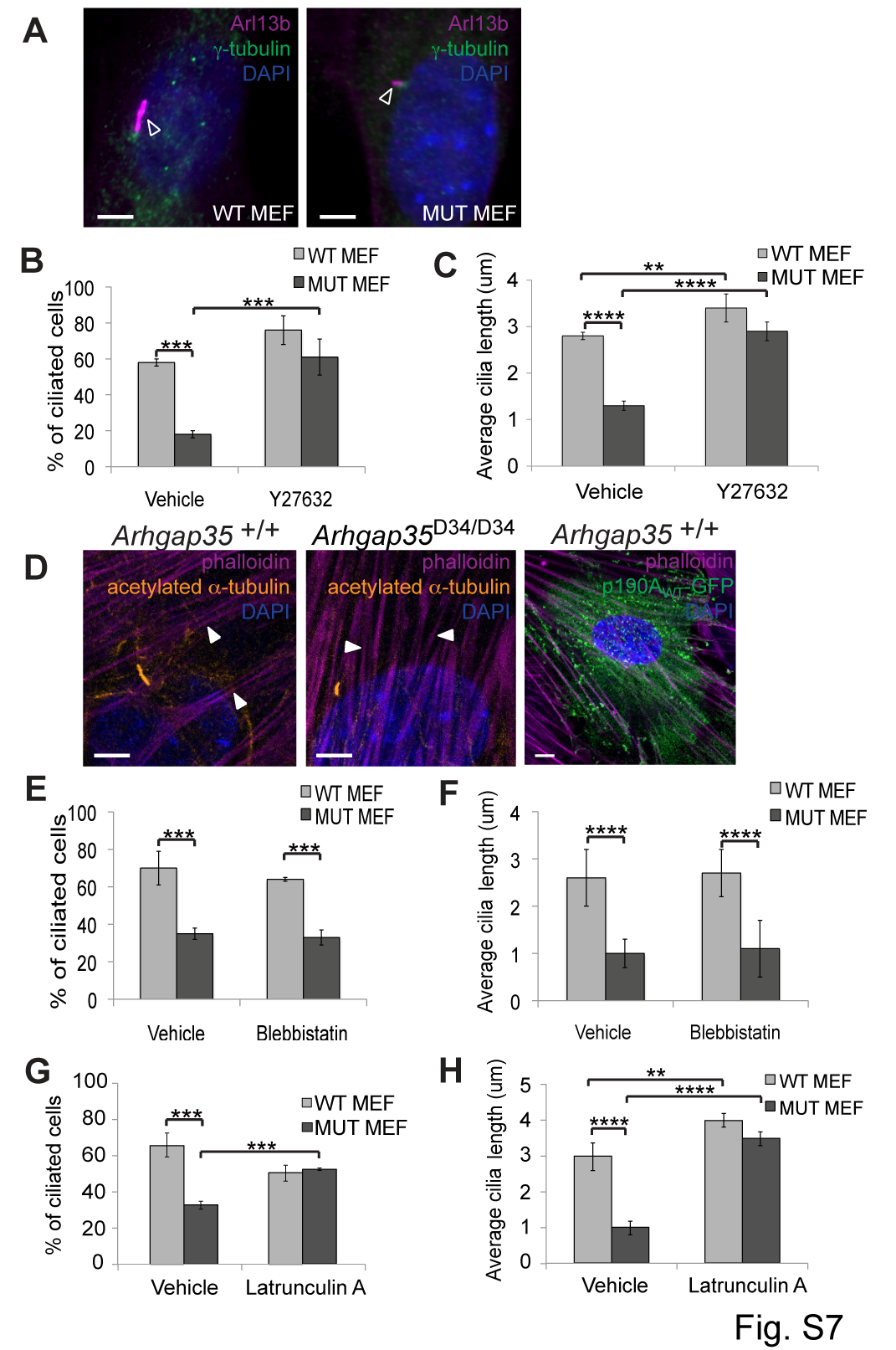

Supplement: S7 Fig — (A) Immunofluorescence staining for γ-tubulin (basal body) and Arl13b (axoneme) reveal a defect in cilia elongation in Arhgap35D34/D34 mouse embryonic fibroblasts compared to control. (B-C) Treatment with the ROCK1/2 inhibitor, Y27632, rescues the defects in cilia number (B) and cilia length (C) in Arhgap35D34/D34 mouse embryonic fibroblasts. (D) Immunofluorescence for phalloidin and acetylated α-tubulin in control and Arhgap35D34/D34 mouse embryonic fibroblasts shows a normal F-actin cytoskeleton in ciliated cells. Staining for phalloidin in mouse embryonic fibroblasts overexpressing full length p190AWT-GFP shows no noticeable effect on F-actin organization. (E-F) Treatment with the myosin II inhibitor Blebbistatin does not rescue the defects in cilia number (D) and cilia length (E) in Arhgap35D34/D34 mouse embryonic fibroblasts. (G-H) Treatment with the actin polymerization inhibitor Latrunculin A rescues the defects in in cilia number (G) and cilia length (H) in Arhgap35D34/D34 mouse embryonic fibroblasts. *p<0.05, **p<0.01, ***p<0.005, ****p<0.001 (one-way ANOVA) (TIF) [file pgen.1005785.s007.tif]
